# Crystal structures of methyl (*E*)-3-(2-chloro­phen­yl)-2-({2-[(*E*)-2-nitro­vin­yl]phen­oxy}meth­yl)acrylate and methyl (*E*)-2-({4-chloro-2-[(*E*)-2-nitro­vin­yl]phen­oxy}meth­yl)-3-(2-chloro­phen­yl)acrylate

**DOI:** 10.1107/S2056989016001493

**Published:** 2016-01-30

**Authors:** G. Vimala, N. Poomathi, P. T. Perumal, A. SubbiahPandi

**Affiliations:** a Department of Physics, Presidency College (Autonomous), Chennai 600 005, India; b Organic Chemistry, CSIR – Central Leather Research Institute, Adyar, Chennai 600 020, India

**Keywords:** crystal structure, 2-cyano­acrylates, phenyl acrylates, cinnamic acid derivatives, C—H⋯O hydrogen bonding

## Abstract

The title compounds, C_19_H_16_ClNO_5_, (I), and C_19_H_15_Cl_2_NO_5_, (II), both crystallize in the monoclinic space group *P*2_1_/*n*. They differ essentially in the orientation of the methyl acetate group, with the C=O bond directed towards the NO_2_ group in (I) but away from it in (II). In compound (I), the mean plane of the methyl acrylate unit is planar, with a maximum deviation of 0.0044 (2) Å for the methyl C atom, while in (II) this deviation is 0.0147 Å. The inter­planar angles between the two aromatic rings are 74.87 (9) and 75.65 (2)° for compounds (I) and (II), respectively. In both compounds, the methyl acrylate and nitro­vinyl groups each adopt an *E* conformation about the C=C bond. In the crystal of (I), mol­ecules are linked by C—H⋯O hydrogen bonds forming chains along the *b* axis. The chains are linked *via* C—H⋯Cl hydrogen bonds, forming sheets parallel to the *ab* plane. The sheets are linked *via* C—H⋯π inter­actions, forming a three-dimensional structure. In the crystal of (II), mol­ecules are linked by pairs of C—H⋯O hydrogen bonds, forming inversion dimers with an *R*_2_^2^(30) ring motif. The dimers are linked *via* C—H⋯O hydrogen bonds, forming sheets parallel to the *ac* plane and enclosing *R*_4_^4^(28) ring motifs. The sheets are linked *via* parallel slipped π–π inter­actions (inter­centroid distances are both *ca* 3.86 Å), forming a three-dimensional structure.

## Abstract 2

In the title compounds, (I) and (II), both of which crystallize in the monoclinic space group *P*2_1_/*n*, the methyl acrylate and nitro­vinyl units are relatively planar with an *E* conformation about the C=C bonds. The two aromatic rings are inclined to one another by 74.87 (9) and 75.65 (2)° for compounds (I) and (II), respectively. In the crystal of (I), chains along the *b* axis are formed *via* C—H⋯O hydrogen bonds. In the crystal of (II), mol­ecules are linked by C—H⋯O hydrogen bonds, forming sheets parallel to the *ac* plane.

## Chemical context   

Recently, 2-cyano­acrylates have been used extensively as agrochemicals because of their unique mechanism of action and good environmental profiles (Govindan *et al.*, 2011 ▸). Phenyl acrylates and their derivatives are important compounds because of their agrochemical and medical applications (De Fraine & Martin, 1991 ▸). Cinnamic acid derivatives have received attention in medicinal research as traditional as well as recently synthetic anti­tumor agents (De *et al.*, 2011 ▸). They also possess significant anti­bacterial activity against *Staphylococcus aureus* (Xiao *et al.*, 2008 ▸). In addition, different substitutions on the basic moiety lead to various pharmacological activities, such as anti-oxidant, hepatoprotective, anxiolytic, insect repellent, anti­diabetic and anti­cholesterolemic (Sharma, 2011 ▸). Against this background, the title compounds were synthesized and we report herein on their crystal structures.

## Structural commentary   

**Figure 1:**
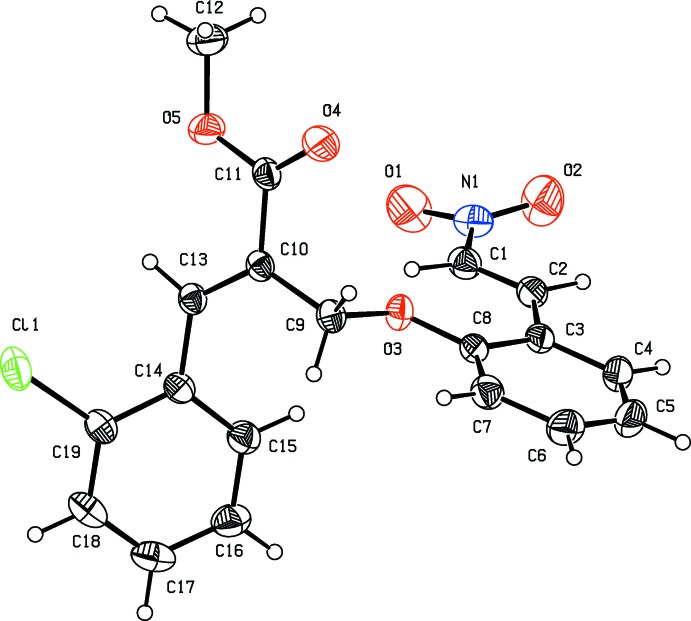
The mol­ecular structure of compound (I), showing the atom labelling. Displacement ellipsoids are drawn at the 30% probability level.

The title compounds, (I) and (II), crystallized in the monoclinic space group *P*2_1_/*n* with *Z* = 4; their mol­ecular structures are illustrated in Figs. 1 ▸ and 2 ▸, respectively. In compound (I), the methyl acrylate unit is essentially planar, with a maximum deviation of 0.0044 (2) Å for atom C12, and forms dihedral angles of 84.04 (9) and 50.23 (9)° with the benzene rings (C3–C8) and (C14–C19), respectively. Likewise, in compound (II), the methyl acrylate unit is essentially planar, with a maximum deviation of 0.0147 (2) Å for atom C12, and forms dihedral angle of 73.20 (9) and 42.81 (9)° with benzene rings (C3–C8) and (C14–C19), respectively. In compound (I), the rings (C3–C8) and (C14–C19) are almost normal to one another, making a dihedral angle of 74.87 (9)°. In the case of compound (II), the corresponding dihedral angle is 75.65 (2)°. The title mol­ecules exhibit structural similarities with the related structure, (*Z*)-methyl 3-(2,4-di­chloro­phen­yl)-2-[(2-formyl­phen­oxy)meth­yl]acrylate (Gangadharan *et al.*, 2011 ▸).
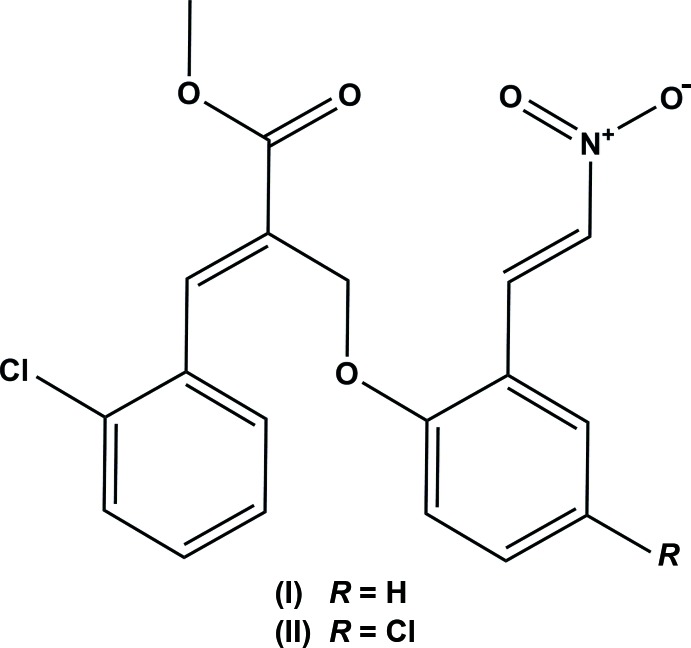


The methyl acrylate moieties adopt an extended conformation, as is evident from the torsion angles O4—C11—C10—C13 = 170.6 (2)°, O5—C11—C10—C13 = −8.5 (2)°, C9—C10—C11—O4 = −5.5 (2)° and C9—C10—C11—O5 = 175.5 (1)° for compound (I), while the corresponding angles in compound (II) are −2.9 (5), 177.7 (3), 173.0 (3) and −6.3 (4)°, respectively. The extended conformation is supported by the fact that the bond angles involving the carbonyl O atoms are invariably enlarged (Schweizer & Dunitz, 1982 ▸).

The significant difference in the bond lengths O5—C11 and O5—C12, which are 1.324 (2) and 1.444 (2) Å, respectively, for compound (I), and 1.328 (4) and 1.440 (4) Å, respectively, for compound (II), can be attributed to a partial contribution from the O^−^—C=O^+^—C resonance structures of the O5—C11(=O4)—C10 group (Merlino *et al.*, 1971 ▸). This feature, commonly observed for the carb­oxy­lic ester group of substit­uents in various compounds gives average values of 1.340 and 1.447 Å, respectively (Varghese *et al.*, 1986 ▸).

In both compounds, the nitro­vinyl groups [C2=C1—N1(O1,O2)], have an *E* conformation about the C2=C1 bond. In (I), its mean plane makes a dihedral angle of 2.025 (9)° with the benzene ring (C3–C8) to which it is attached, while in compound (II), the corresponding dihedral angle is much larger, at 14.78 (16) °.

## Supra­molecular features   

**Figure 3:**
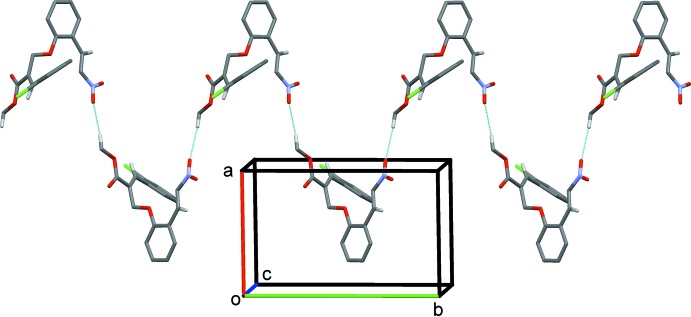
A partial view of the crystal structure of compound (I), showing the hydrogen-bonded (dashed lines) zigzag chains propagating along [010]; see Table 1 ▸.

**Figure 4:**
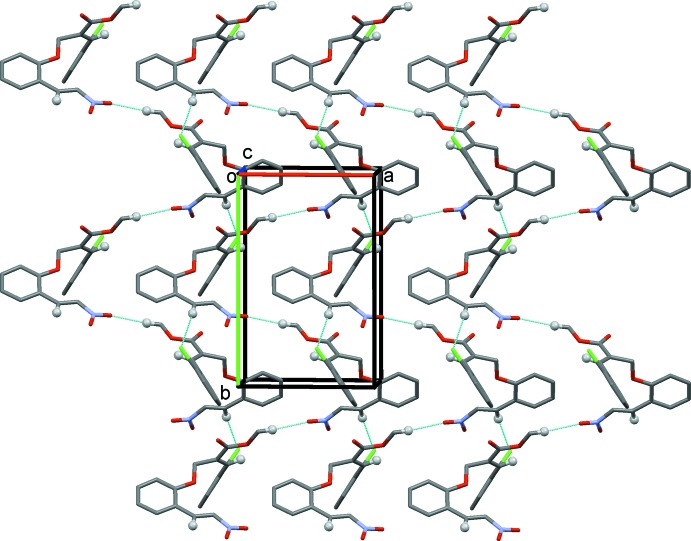
The crystal packing of compound (I), viewed along the *c* axis. The hydrogen bonds are shown as dashed lines (see Table 1 ▸).

In the crystal of (I), adjacent mol­ecules are linked by C—H⋯O hydrogen bonds forming chains along the *b-*axis direction (Table 1 ▸ and Fig. 3 ▸). The chains are linked *via* C—H⋯Cl hydrogen bonds, forming sheets parallel to the *ab* plane (Fig. 4 ▸ and Table 1 ▸). The sheets are linked *via* C—H⋯π inter­actions, forming a three-dimensional structure (Table 1 ▸).

**Figure 5:**
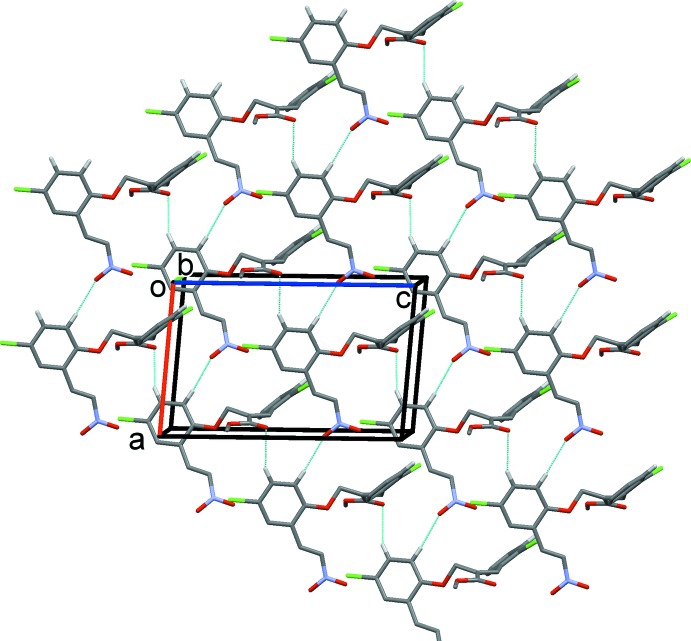
A partial view of the crystal packing of compound (II), viewed along the *b* axis. The hydrogen bonds are shown as dashed lines (see Table 2 ▸).

**Figure 6:**
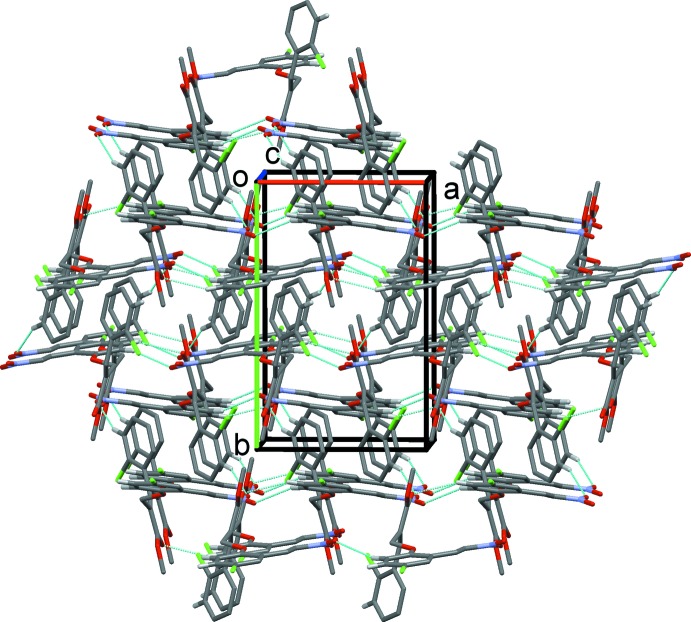
The crystal packing of compound (II), viewed along the *c* axis. The hydrogen bonds are shown as dashed lines (see Table 1 ▸).

In compound (II), mol­ecules are linked by pairs of C—H⋯O hydrogen bonds, forming inversion dimers enclosing an 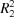
(30) ring motif (Table 2 ▸ and Fig. 5 ▸). The dimers are linked by further C—H⋯O hydrogen bonds, forming sheets parallel to the *ac* plane and enclosing 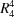
(28) ring motifs (Table 2 ▸ and Fig. 5 ▸). The sheets are linked *via* slipped parallel π–π inter­actions, forming a three-dimensional structure, Fig. 6 ▸ [*Cg*1⋯*Cg*1^i^ = 3.863 (2) Å, inter-planar distance = 3.487 (1) Å, slippage 1.662 Å; *Cg*1 is the centroid of ring C3–C8; symmetry code: (i) −*x* + 1, −*y*, *z* + 1, and *Cg*2⋯*Cg*2^ii^ = 3.861 (2) Å, inter-planar distance = 3.506 (2) Å, slippage = 1.617 Å; *Cg*2 is the centroid of ring C14–C19; symmetry code: (ii) −*x* + 1, −*y*, −*z* + 2].

## Database survey   

A search of the Cambridge Structural Database (CSD, Version 5.37, November 2015; Groom & Allen, 2014 ▸) for the substructure methyl (*E*)-2-(phen­oxy­meth­yl)-3-phenyl­acrylate gave 12 hits. There is a great variety in the dihedral angle involving the two aromatic rings; from a minimum of *ca* 47.2° in (*E*)-methyl 2-({2-eth­oxy-6-[(*E*)-(hy­droxy­imino)­meth­yl]phen­oxy}meth­yl)-3-phenyl­acrylate (CSD code: ZARDAT; Govindan *et al.*, 2012 ▸) to a maximum of *ca* 88.4° in methyl (*E*)-2-[(2-nitro­phen­oxy)meth­yl]-3-phenyl­acrylate (CSD code: PAWFIE; Anuradha *et al.*, 2012 ▸). In the title compounds, this dihedral angle is 74.87 (9)° in (I) and 75.65 (2)° in (II).

## Synthesis and crystallization   

The title compounds were prepared in a similar manner using a mixture of methyl (*E*)-3-(2-chloro­phen­yl)-2-{[2-(2,2-di­cyano­vin­yl)phen­oxy]meth­yl}acrylate (1 mmol) for compound (I), and methyl (*E*)-2-{[4-chloro-2-(2,2-di­cyano­vin­yl)phen­oxy]meth­yl}-3-(2-chloro­phen­yl)acrylate (1 mol) for compound (II), dissolved in nitro­methane (5 mol) in toluene (3 ml) with a catalytic amount of cinchona alkaloid (0.005 mmol %). The resulting solutions were stirred for 4 h at room temperature. The consumption of the starting materials was monitored by TLC. After completion of the reaction, DMAP (0.020 mol %) and di-*tert*-butyl dicarbonate (1.2 equiv) were added and the solutions of the corresponding crude products were stirred at 318–323 K for 2 h, followed by TLC (20% EtOAc and petroleum ether). The solvents were removed under reduced pressure and the residues purified by column chromatography on silica gel (3:97%, ethyl­acetate and petroleum ether) to afford pure products. The purified compounds were recrystallized from ethanol, by slow evaporation of the solvent, yielding block-like crystals of compounds (I) and (II), suitable for X ray diffraction analysis.

## Refinement   

Crystal data, data collection and structure refinement details are summarized in Table 3 ▸. The C-bound H atoms were positioned geometrically (C—H = 0.93–0.97 Å) and allowed to ride on their parent atoms, with *U*_iso_(H) = 1.5*U*_eq_(C-meth­yl) and 1.2*U*_eq_(C) for other H atoms.

## Supplementary Material

Crystal structure: contains datablock(s) global, I, II. DOI: 10.1107/S2056989016001493/su5265sup1.cif

Structure factors: contains datablock(s) I. DOI: 10.1107/S2056989016001493/su5265Isup2.hkl

Structure factors: contains datablock(s) II. DOI: 10.1107/S2056989016001493/su5265IIsup3.hkl

Supporting information file. DOI: 10.1107/S2056989016001493/su5265Isup4.cml

Supporting information file. DOI: 10.1107/S2056989016001493/su5265IIsup5.cml

CCDC references: 1449405, 1449404

Additional supporting information:  crystallographic information; 3D view; checkCIF report

## Figures and Tables

**Figure 2:**
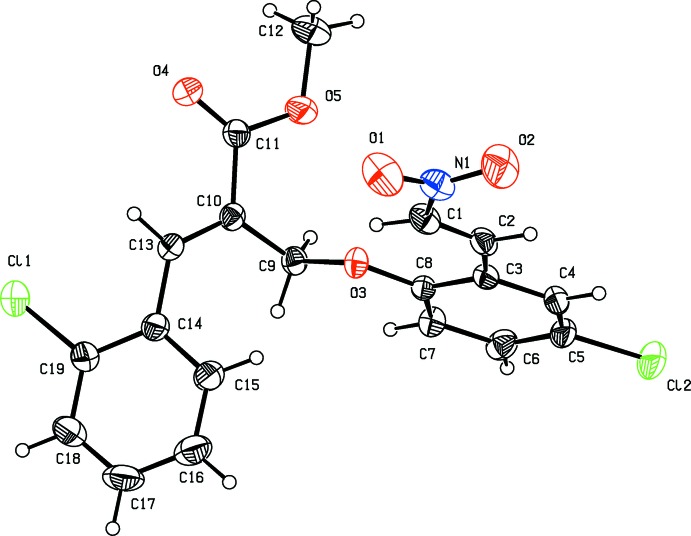
The mol­ecular structure of compound (II), showing the atom labelling. Displacement ellipsoids are drawn at the 30% probability level.

**Table 1:** Hydrogen-bond geometry (Å, °) for (I) *Cg*2 is the centroid of the C14–C19 ring.

*D*—H⋯*A*	*D*—H	H⋯*A*	*D*⋯*A*	*D*—H⋯*A*
C12—H12*A*⋯O1^i^	0.96	2.45	3.406 (2)	172
C2—H2⋯Cl1^ii^	0.93	2.85	3.7515 (16)	165
C13—H13⋯*Cg*2^iii^	0.93	2.91	3.5828 (16)	130

Symmetry codes: (i) 
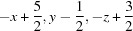
; (ii) 
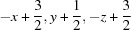
; (iii) 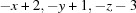.

**Table 2:** Hydrogen-bond geometry (Å, °) for (II)

*D*—H⋯*A*	*D*—H	H⋯*A*	*D*⋯*A*	*D*—H⋯*A*
C6—H6⋯O4^i^	0.93	2.56	3.371 (4)	146
C7—H7⋯O2^ii^	0.93	2.58	3.476 (4)	161
C18—H18⋯O1^iii^	0.93	2.60	3.485 (5)	160

Symmetry codes: (i) 
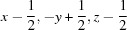
; (ii) 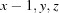; (iii) 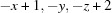.

**Table 3:** Experimental details

	(I)	(II)
Crystal data		
Chemical formula	C_19_H_16_ClNO_5_	C_19_H_15_Cl_2_NO_5_
*M*_r_	373.78	408.22
Crystal system, space group	Monoclinic, *P*2_1_/*n*	Monoclinic, *P*2_1_/*n*
Temperature (K)	293	293
*a*, *b*, *c* (Å)	9.0152 (3), 13.6579 (4), 14.6366 (4)	9.2372 (3), 14.5027 (5), 14.4830 (5)
β (°)	102.176 (1)	94.521 (2)
*V* (Å^3^)	1761.64 (9)	1934.17 (11)
*Z*	4	4
Radiation type	Mo *K*α	Mo *K*α
μ (mm^−1^)	0.25	0.37
Crystal size (mm)	0.27 × 0.24 × 0.18	0.28 × 0.22 × 0.19

Data collection		
Diffractometer	Bruker Kappa APEXII CCD	Bruker Kappa APEXII CCD
Absorption correction	Multi-scan (*SADABS*; Bruker, 2008 ▸)	Multi-scan (*SADABS*; Bruker, 2008 ▸)
*T*_min_, *T*_max_	0.935, 0.935	0.942, 0.961
No. of measured, independent and observed [*I* > 2σ(*I*)] reflections	15968, 4365, 3186	12108, 3481, 2382
*R*_int_	0.019	0.029
(sin θ/λ)_max_ (Å^−1^)	0.667	0.600

Refinement		
*R*[*F* ^2^ > 2σ(*F* ^2^)], *wR*(*F* ^2^), *S*	0.040, 0.109, 1.04	0.055, 0.143, 1.04
No. of reflections	4365	3481
No. of parameters	236	245
H-atom treatment	H-atom parameters constrained	H-atom parameters constrained
Δρ_max_, Δρ_min_ (e Å^−3^)	0.23, −0.23	0.57, −0.31

Computer programs: *APEX2* and *SAINT* (Bruker, 2008 ▸), *SHELXS97* (Sheldrick, 2008 ▸), *SHELXL2014* (Sheldrick, 2015 ▸), *PLATON* (Spek, 2009 ▸) and *Mercury* (Macrae *et al.*, 2008 ▸).

## supplementary crystallographic information

### (I) Methyl (E)-3-(2-chlorophenyl)-2-({2-[(E)-2-nitrovinyl]phenoxy}methyl)acrylate Crystal data

**Table:**

C_19_H_16_ClNO_5_	*F*(000) = 776
*M**_r_* = 373.78	*D*_x_ = 1.409 Mg m^−^^3^
Monoclinic, *P*2_1_/*n*	Mo *K*α radiation, λ = 0.71073 Å
*a* = 9.0152 (3) Å	Cell parameters from 2595 reflections
*b* = 13.6579 (4) Å	θ = 2.1–25.0°
*c* = 14.6366 (4) Å	µ = 0.25 mm^−^^1^
β = 102.176 (1)°	*T* = 293 K
*V* = 1761.64 (9) Å^3^	Block, colourless
*Z* = 4	0.27 × 0.24 × 0.18 mm

### (I) Methyl (E)-3-(2-chlorophenyl)-2-({2-[(E)-2-nitrovinyl]phenoxy}methyl)acrylate Data collection

**Table:**

Bruker Kappa APEXII CCD diffractometer	4365 independent reflections
Radiation source: fine-focus sealed tube	3186 reflections with *I* > 2σ(*I*)
Graphite monochromator	*R*_int_ = 0.019
ω and φ scans	θ_max_ = 28.3°, θ_min_ = 2.1°
Absorption correction: multi-scan (*SADABS*; Bruker, 2008)	*h* = −9→12
*T*_min_ = 0.935, *T*_max_ = 0.935	*k* = −9→18
15968 measured reflections	*l* = −19→19

### (I) Methyl (E)-3-(2-chlorophenyl)-2-({2-[(E)-2-nitrovinyl]phenoxy}methyl)acrylate Refinement

**Table:**

Refinement on *F*^2^	0 restraints
Least-squares matrix: full	Hydrogen site location: inferred from neighbouring sites
*R*[*F*^2^ > 2σ(*F*^2^)] = 0.040	H-atom parameters constrained
*wR*(*F*^2^) = 0.109	*w* = 1/[σ^2^(*F*_o_^2^) + (0.0464*P*)^2^ + 0.4238*P*] where *P* = (*F*_o_^2^ + 2*F*_c_^2^)/3
*S* = 1.04	(Δ/σ)_max_ = 0.001
4365 reflections	Δρ_max_ = 0.23 e Å^−^^3^
236 parameters	Δρ_min_ = −0.23 e Å^−^^3^

### (I) Methyl (E)-3-(2-chlorophenyl)-2-({2-[(E)-2-nitrovinyl]phenoxy}methyl)acrylate Special details

**Table:**

Geometry. All e.s.d.'s (except the e.s.d. in the dihedral angle between two l.s. planes) are estimated using the full covariance matrix. The cell e.s.d.'s are taken into account individually in the estimation of e.s.d.'s in distances, angles and torsion angles; correlations between e.s.d.'s in cell parameters are only used when they are defined by crystal symmetry. An approximate (isotropic) treatment of cell e.s.d.'s is used for estimating e.s.d.'s involving l.s. planes.

### (I) Methyl (E)-3-(2-chlorophenyl)-2-({2-[(E)-2-nitrovinyl]phenoxy}methyl)acrylate Fractional atomic coordinates and isotropic or equivalent isotropic displacement parameters (Å^2^)

**Table:**

	*x*	*y*	*z*	*U*_iso_*/*U*_eq_	
Cl1	0.94245 (6)	0.33362 (3)	1.09845 (3)	0.06629 (16)	
O1	1.02396 (17)	0.69069 (14)	0.66665 (11)	0.0909 (5)	
O2	0.88624 (18)	0.72882 (13)	0.53405 (12)	0.0931 (5)	
O3	0.63790 (11)	0.47698 (8)	0.70425 (7)	0.0448 (3)	
O4	0.81629 (15)	0.27705 (11)	0.67330 (8)	0.0732 (4)	
O5	1.02681 (12)	0.29776 (9)	0.78215 (8)	0.0572 (3)	
N1	0.90607 (17)	0.68625 (11)	0.60846 (11)	0.0562 (4)	
C1	0.78561 (18)	0.62664 (12)	0.63151 (11)	0.0480 (4)	
H1	0.8059	0.5842	0.6823	0.058*	
C2	0.64741 (18)	0.63313 (11)	0.57985 (10)	0.0454 (4)	
H2	0.6373	0.6754	0.5291	0.054*	
C3	0.50814 (17)	0.58452 (11)	0.58984 (9)	0.0420 (3)	
C4	0.3720 (2)	0.61704 (14)	0.53395 (11)	0.0553 (4)	
H4	0.3738	0.6682	0.4923	0.066*	
C5	0.2351 (2)	0.57515 (15)	0.53901 (13)	0.0645 (5)	
H5	0.1456	0.5985	0.5018	0.077*	
C6	0.2317 (2)	0.49857 (15)	0.59942 (13)	0.0624 (5)	
H6	0.1392	0.4702	0.6029	0.075*	
C7	0.36441 (18)	0.46297 (13)	0.65535 (11)	0.0512 (4)	
H7	0.3610	0.4105	0.6953	0.061*	
C8	0.50191 (17)	0.50605 (11)	0.65131 (9)	0.0404 (3)	
C9	0.64005 (16)	0.39327 (11)	0.76435 (10)	0.0415 (3)	
H9A	0.5931	0.3375	0.7285	0.050*	
H9B	0.5844	0.4074	0.8127	0.050*	
C10	0.80192 (16)	0.37129 (10)	0.80709 (9)	0.0371 (3)	
C11	0.88048 (18)	0.31119 (11)	0.74673 (10)	0.0421 (3)	
C12	1.1079 (2)	0.23635 (15)	0.72892 (14)	0.0658 (5)	
H12A	1.2107	0.2281	0.7627	0.099*	
H12B	1.1078	0.2666	0.6697	0.099*	
H12C	1.0593	0.1736	0.7190	0.099*	
C13	0.87143 (16)	0.39681 (10)	0.89424 (9)	0.0387 (3)	
H13	0.9701	0.3744	0.9152	0.046*	
C14	0.80700 (16)	0.45658 (11)	0.95956 (10)	0.0401 (3)	
C15	0.72385 (19)	0.54180 (12)	0.93057 (11)	0.0503 (4)	
H15	0.7068	0.5599	0.8680	0.060*	
C16	0.6666 (2)	0.59956 (14)	0.99228 (13)	0.0595 (4)	
H16	0.6118	0.6558	0.9713	0.071*	
C17	0.6908 (2)	0.57362 (15)	1.08512 (13)	0.0616 (5)	
H17	0.6509	0.6120	1.1266	0.074*	
C18	0.7739 (2)	0.49116 (14)	1.11704 (11)	0.0566 (4)	
H18	0.7907	0.4740	1.1799	0.068*	
C19	0.83196 (18)	0.43422 (11)	1.05485 (10)	0.0448 (4)	

### (I) Methyl (E)-3-(2-chlorophenyl)-2-({2-[(E)-2-nitrovinyl]phenoxy}methyl)acrylate Atomic displacement parameters (Å^2^)

**Table:**

	*U*^11^	*U*^22^	*U*^33^	*U*^12^	*U*^13^	*U*^23^
Cl1	0.0968 (4)	0.0562 (3)	0.0402 (2)	0.0052 (2)	0.0017 (2)	0.00662 (18)
O1	0.0637 (9)	0.1316 (15)	0.0765 (10)	−0.0259 (9)	0.0127 (8)	−0.0043 (10)
O2	0.0839 (10)	0.0980 (12)	0.1021 (12)	−0.0038 (9)	0.0300 (9)	0.0478 (10)
O3	0.0423 (6)	0.0458 (6)	0.0437 (5)	0.0003 (5)	0.0033 (4)	0.0125 (5)
O4	0.0721 (8)	0.0937 (10)	0.0474 (7)	0.0174 (7)	−0.0020 (6)	−0.0269 (7)
O5	0.0500 (7)	0.0665 (8)	0.0533 (7)	0.0096 (6)	0.0071 (5)	−0.0167 (6)
N1	0.0609 (10)	0.0516 (8)	0.0611 (9)	0.0006 (7)	0.0237 (8)	−0.0041 (7)
C1	0.0572 (10)	0.0432 (8)	0.0456 (8)	0.0012 (7)	0.0154 (7)	0.0017 (7)
C2	0.0615 (10)	0.0366 (8)	0.0392 (7)	0.0090 (7)	0.0131 (7)	0.0024 (6)
C3	0.0519 (9)	0.0397 (8)	0.0332 (7)	0.0087 (6)	0.0060 (6)	−0.0041 (6)
C4	0.0617 (11)	0.0563 (10)	0.0435 (8)	0.0161 (8)	0.0007 (7)	0.0005 (7)
C5	0.0525 (11)	0.0774 (13)	0.0558 (10)	0.0168 (9)	−0.0065 (8)	−0.0059 (10)
C6	0.0430 (9)	0.0790 (13)	0.0621 (11)	−0.0005 (9)	0.0038 (8)	−0.0137 (10)
C7	0.0489 (9)	0.0589 (10)	0.0447 (8)	−0.0015 (8)	0.0077 (7)	−0.0020 (7)
C8	0.0437 (8)	0.0436 (8)	0.0325 (7)	0.0051 (6)	0.0045 (6)	−0.0055 (6)
C9	0.0457 (8)	0.0394 (8)	0.0387 (7)	−0.0033 (6)	0.0074 (6)	0.0042 (6)
C10	0.0456 (8)	0.0313 (7)	0.0345 (7)	−0.0012 (6)	0.0089 (6)	0.0048 (5)
C11	0.0532 (9)	0.0383 (8)	0.0341 (7)	0.0024 (6)	0.0079 (6)	0.0036 (6)
C12	0.0612 (11)	0.0704 (12)	0.0683 (11)	0.0132 (9)	0.0196 (9)	−0.0120 (10)
C13	0.0440 (8)	0.0355 (7)	0.0362 (7)	0.0002 (6)	0.0075 (6)	0.0045 (6)
C14	0.0433 (8)	0.0398 (8)	0.0373 (7)	−0.0053 (6)	0.0089 (6)	−0.0030 (6)
C15	0.0577 (10)	0.0482 (9)	0.0452 (8)	0.0031 (7)	0.0110 (7)	−0.0020 (7)
C16	0.0591 (11)	0.0536 (10)	0.0671 (11)	0.0078 (8)	0.0165 (9)	−0.0100 (9)
C17	0.0613 (11)	0.0664 (12)	0.0635 (11)	−0.0070 (9)	0.0277 (9)	−0.0227 (9)
C18	0.0688 (11)	0.0644 (11)	0.0405 (8)	−0.0168 (9)	0.0203 (8)	−0.0109 (8)
C19	0.0515 (9)	0.0438 (8)	0.0386 (7)	−0.0101 (7)	0.0082 (6)	−0.0037 (6)

### (I) Methyl (E)-3-(2-chlorophenyl)-2-({2-[(E)-2-nitrovinyl]phenoxy}methyl)acrylate Geometric parameters (Å, º)

**Table:**

Cl1—C19	1.7377 (17)	C7—H7	0.9300
O1—N1	1.2152 (19)	C9—C10	1.492 (2)
O2—N1	1.214 (2)	C9—H9A	0.9700
O3—C8	1.3639 (17)	C9—H9B	0.9700
O3—C9	1.4403 (17)	C10—C13	1.3431 (19)
O4—C11	1.2022 (18)	C10—C11	1.490 (2)
O5—C11	1.3244 (19)	C12—H12A	0.9600
O5—C12	1.444 (2)	C12—H12B	0.9600
N1—C1	1.453 (2)	C12—H12C	0.9600
C1—C2	1.317 (2)	C13—C14	1.467 (2)
C1—H1	0.9300	C13—H13	0.9300
C2—C3	1.455 (2)	C14—C19	1.399 (2)
C2—H2	0.9300	C14—C15	1.401 (2)
C3—C4	1.396 (2)	C15—C16	1.379 (2)
C3—C8	1.408 (2)	C15—H15	0.9300
C4—C5	1.376 (3)	C16—C17	1.376 (3)
C4—H4	0.9300	C16—H16	0.9300
C5—C6	1.374 (3)	C17—C18	1.378 (3)
C5—H5	0.9300	C17—H17	0.9300
C6—C7	1.388 (2)	C18—C19	1.381 (2)
C6—H6	0.9300	C18—H18	0.9300
C7—C8	1.385 (2)		
			
C8—O3—C9	118.21 (11)	H9A—C9—H9B	108.5
C11—O5—C12	116.44 (13)	C13—C10—C11	121.41 (13)
O2—N1—O1	123.12 (17)	C13—C10—C9	124.41 (13)
O2—N1—C1	120.05 (16)	C11—C10—C9	114.05 (12)
O1—N1—C1	116.83 (15)	O4—C11—O5	123.24 (14)
C2—C1—N1	119.47 (15)	O4—C11—C10	122.99 (15)
C2—C1—H1	120.3	O5—C11—C10	113.77 (12)
N1—C1—H1	120.3	O5—C12—H12A	109.5
C1—C2—C3	130.07 (15)	O5—C12—H12B	109.5
C1—C2—H2	115.0	H12A—C12—H12B	109.5
C3—C2—H2	115.0	O5—C12—H12C	109.5
C4—C3—C8	117.89 (15)	H12A—C12—H12C	109.5
C4—C3—C2	117.83 (15)	H12B—C12—H12C	109.5
C8—C3—C2	124.27 (13)	C10—C13—C14	126.43 (14)
C5—C4—C3	121.58 (17)	C10—C13—H13	116.8
C5—C4—H4	119.2	C14—C13—H13	116.8
C3—C4—H4	119.2	C19—C14—C15	116.54 (14)
C6—C5—C4	119.57 (16)	C19—C14—C13	121.72 (14)
C6—C5—H5	120.2	C15—C14—C13	121.67 (13)
C4—C5—H5	120.2	C16—C15—C14	121.82 (16)
C5—C6—C7	120.82 (18)	C16—C15—H15	119.1
C5—C6—H6	119.6	C14—C15—H15	119.1
C7—C6—H6	119.6	C17—C16—C15	119.73 (17)
C8—C7—C6	119.63 (17)	C17—C16—H16	120.1
C8—C7—H7	120.2	C15—C16—H16	120.1
C6—C7—H7	120.2	C16—C17—C18	120.45 (16)
O3—C8—C7	123.89 (14)	C16—C17—H17	119.8
O3—C8—C3	115.62 (13)	C18—C17—H17	119.8
C7—C8—C3	120.49 (14)	C17—C18—C19	119.40 (16)
O3—C9—C10	107.56 (11)	C17—C18—H18	120.3
O3—C9—H9A	110.2	C19—C18—H18	120.3
C10—C9—H9A	110.2	C18—C19—C14	122.04 (16)
O3—C9—H9B	110.2	C18—C19—Cl1	118.07 (13)
C10—C9—H9B	110.2	C14—C19—Cl1	119.87 (12)
			
O2—N1—C1—C2	−12.4 (2)	C12—O5—C11—O4	−1.8 (2)
O1—N1—C1—C2	167.54 (17)	C12—O5—C11—C10	177.29 (14)
N1—C1—C2—C3	−177.77 (14)	C13—C10—C11—O4	170.56 (15)
C1—C2—C3—C4	169.58 (16)	C9—C10—C11—O4	−5.5 (2)
C1—C2—C3—C8	−11.3 (3)	C13—C10—C11—O5	−8.5 (2)
C8—C3—C4—C5	0.8 (2)	C9—C10—C11—O5	175.47 (12)
C2—C3—C4—C5	−179.97 (15)	C11—C10—C13—C14	179.10 (13)
C3—C4—C5—C6	−0.9 (3)	C9—C10—C13—C14	−5.3 (2)
C4—C5—C6—C7	0.0 (3)	C10—C13—C14—C19	139.80 (16)
C5—C6—C7—C8	1.0 (3)	C10—C13—C14—C15	−43.5 (2)
C9—O3—C8—C7	3.4 (2)	C19—C14—C15—C16	−1.4 (2)
C9—O3—C8—C3	−176.32 (12)	C13—C14—C15—C16	−178.25 (15)
C6—C7—C8—O3	179.22 (14)	C14—C15—C16—C17	0.0 (3)
C6—C7—C8—C3	−1.0 (2)	C15—C16—C17—C18	1.0 (3)
C4—C3—C8—O3	179.92 (13)	C16—C17—C18—C19	−0.4 (3)
C2—C3—C8—O3	0.8 (2)	C17—C18—C19—C14	−1.1 (2)
C4—C3—C8—C7	0.2 (2)	C17—C18—C19—Cl1	177.47 (13)
C2—C3—C8—C7	−178.99 (14)	C15—C14—C19—C18	2.0 (2)
C8—O3—C9—C10	174.75 (12)	C13—C14—C19—C18	178.82 (14)
O3—C9—C10—C13	100.78 (15)	C15—C14—C19—Cl1	−176.57 (12)
O3—C9—C10—C11	−83.34 (14)	C13—C14—C19—Cl1	0.3 (2)

### (I) Methyl (E)-3-(2-chlorophenyl)-2-({2-[(E)-2-nitrovinyl]phenoxy}methyl)acrylate Hydrogen-bond geometry (Å, º)

Cg2 is the centroid of the C14–C19 ring.

**Table:**

*D*—H···*A*	*D*—H	H···*A*	*D*···*A*	*D*—H···*A*
C12—H12*A*···O1^i^	0.96	2.45	3.406 (2)	172
C2—H2···Cl1^ii^	0.93	2.85	3.7515 (16)	165
C13—H13···*Cg*2^iii^	0.93	2.91	3.5828 (16)	130

Symmetry codes: (i) −*x*+5/2, *y*−1/2, −*z*+3/2; (ii) −*x*+3/2, *y*+1/2, −*z*+3/2; (iii) −*x*+2, −*y*+1, −*z*−3.

### (II) Methyl (E)-2-({4-chloro-2-[(E)-2-nitrovinyl]phenoxy}methyl)-3-(2-chlorophenyl)acrylate Crystal data

**Table:**

C_19_H_15_Cl_2_NO_5_	*F*(000) = 840
*M**_r_* = 408.22	*D*_x_ = 1.402 Mg m^−^^3^
Monoclinic, *P*2_1_/*n*	Mo *K*α radiation, λ = 0.71073 Å
*a* = 9.2372 (3) Å	Cell parameters from 2355 reflections
*b* = 14.5027 (5) Å	θ = 2.0–25.0°
*c* = 14.4830 (5) Å	µ = 0.37 mm^−^^1^
β = 94.521 (2)°	*T* = 293 K
*V* = 1934.17 (11) Å^3^	Block, yellow
*Z* = 4	0.28 × 0.22 × 0.19 mm

### (II) Methyl (E)-2-({4-chloro-2-[(E)-2-nitrovinyl]phenoxy}methyl)-3-(2-chlorophenyl)acrylate Data collection

**Table:**

Bruker Kappa APEXII CCD diffractometer	3481 independent reflections
Radiation source: fine-focus sealed tube	2382 reflections with *I* > 2σ(*I*)
Graphite monochromator	*R*_int_ = 0.029
ω and φ scans	θ_max_ = 25.2°, θ_min_ = 2.0°
Absorption correction: multi-scan (*SADABS*; Bruker, 2008)	*h* = −11→10
*T*_min_ = 0.942, *T*_max_ = 0.961	*k* = −14→17
12108 measured reflections	*l* = −12→17

### (II) Methyl (E)-2-({4-chloro-2-[(E)-2-nitrovinyl]phenoxy}methyl)-3-(2-chlorophenyl)acrylate Refinement

**Table:**

Refinement on *F*^2^	0 restraints
Least-squares matrix: full	Hydrogen site location: inferred from neighbouring sites
*R*[*F*^2^ > 2σ(*F*^2^)] = 0.055	H-atom parameters constrained
*wR*(*F*^2^) = 0.143	*w* = 1/[σ^2^(*F*_o_^2^) + (0.0555*P*)^2^ + 1.4111*P*] where *P* = (*F*_o_^2^ + 2*F*_c_^2^)/3
*S* = 1.04	(Δ/σ)_max_ < 0.001
3481 reflections	Δρ_max_ = 0.56 e Å^−^^3^
245 parameters	Δρ_min_ = −0.31 e Å^−^^3^

### (II) Methyl (E)-2-({4-chloro-2-[(E)-2-nitrovinyl]phenoxy}methyl)-3-(2-chlorophenyl)acrylate Special details

**Table:**

Geometry. All e.s.d.'s (except the e.s.d. in the dihedral angle between two l.s. planes) are estimated using the full covariance matrix. The cell e.s.d.'s are taken into account individually in the estimation of e.s.d.'s in distances, angles and torsion angles; correlations between e.s.d.'s in cell parameters are only used when they are defined by crystal symmetry. An approximate (isotropic) treatment of cell e.s.d.'s is used for estimating e.s.d.'s involving l.s. planes.

### (II) Methyl (E)-2-({4-chloro-2-[(E)-2-nitrovinyl]phenoxy}methyl)-3-(2-chlorophenyl)acrylate Fractional atomic coordinates and isotropic or equivalent isotropic displacement parameters (Å^2^)

**Table:**

	*x*	*y*	*z*	*U*_iso_*/*U*_eq_	
Cl1	0.17238 (14)	0.12004 (8)	1.07720 (8)	0.0933 (4)	
Cl2	0.41061 (13)	0.09022 (7)	0.31090 (6)	0.0812 (4)	
O1	0.9540 (3)	0.1694 (2)	0.8133 (2)	0.0860 (9)	
O2	0.9968 (3)	0.1892 (2)	0.6721 (2)	0.0870 (9)	
O3	0.46144 (19)	0.15787 (14)	0.71181 (12)	0.0405 (5)	
O4	0.4419 (3)	0.35608 (16)	0.93013 (16)	0.0679 (7)	
O5	0.4219 (3)	0.35463 (15)	0.77613 (15)	0.0611 (6)	
N1	0.9138 (3)	0.17359 (19)	0.7312 (2)	0.0577 (7)	
C1	0.7610 (3)	0.1607 (2)	0.7049 (2)	0.0490 (8)	
H1	0.6942	0.1598	0.7497	0.059*	
C2	0.7171 (3)	0.1501 (2)	0.6157 (2)	0.0457 (7)	
H2	0.7904	0.1516	0.5753	0.055*	
C3	0.5725 (3)	0.13660 (19)	0.5727 (2)	0.0403 (7)	
C4	0.5582 (4)	0.1206 (2)	0.4764 (2)	0.0497 (8)	
H4	0.6407	0.1186	0.4437	0.060*	
C5	0.4247 (4)	0.1082 (2)	0.4307 (2)	0.0500 (8)	
C6	0.3012 (4)	0.1102 (2)	0.4768 (2)	0.0516 (8)	
H6	0.2111	0.1007	0.4449	0.062*	
C7	0.3112 (3)	0.1264 (2)	0.5709 (2)	0.0439 (7)	
H7	0.2271	0.1282	0.6022	0.053*	
C8	0.4440 (3)	0.13990 (18)	0.61909 (18)	0.0356 (7)	
C9	0.3311 (3)	0.1789 (2)	0.75710 (19)	0.0405 (7)	
H9A	0.2733	0.1236	0.7622	0.049*	
H9B	0.2732	0.2241	0.7212	0.049*	
C10	0.3751 (3)	0.2162 (2)	0.85085 (19)	0.0383 (7)	
C11	0.4169 (3)	0.3154 (2)	0.8587 (2)	0.0446 (7)	
C12	0.4565 (5)	0.4514 (2)	0.7742 (3)	0.0754 (11)	
H12A	0.3927	0.4847	0.8114	0.113*	
H12B	0.4448	0.4734	0.7116	0.113*	
H12C	0.5553	0.4605	0.7985	0.113*	
C13	0.3713 (3)	0.1683 (2)	0.9294 (2)	0.0427 (7)	
H13	0.3937	0.2004	0.9842	0.051*	
C14	0.3356 (3)	0.0703 (2)	0.93813 (19)	0.0450 (7)	
C15	0.3945 (4)	0.0031 (2)	0.8832 (2)	0.0538 (8)	
H15	0.4563	0.0207	0.8386	0.065*	
C16	0.3629 (4)	−0.0887 (2)	0.8939 (3)	0.0682 (11)	
H16	0.4031	−0.1326	0.8567	0.082*	
C17	0.2720 (5)	−0.1158 (3)	0.9593 (3)	0.0780 (12)	
H17	0.2501	−0.1779	0.9661	0.094*	
C18	0.2140 (5)	−0.0517 (3)	1.0146 (3)	0.0744 (11)	
H18	0.1525	−0.0702	1.0589	0.089*	
C19	0.2458 (4)	0.0402 (2)	1.0050 (2)	0.0570 (9)	

### (II) Methyl (E)-2-({4-chloro-2-[(E)-2-nitrovinyl]phenoxy}methyl)-3-(2-chlorophenyl)acrylate Atomic displacement parameters (Å^2^)

**Table:**

	*U*^11^	*U*^22^	*U*^33^	*U*^12^	*U*^13^	*U*^23^
Cl1	0.1329 (10)	0.0784 (7)	0.0764 (7)	−0.0237 (7)	0.0577 (7)	−0.0153 (5)
Cl2	0.1300 (9)	0.0795 (7)	0.0346 (5)	−0.0058 (6)	0.0091 (5)	−0.0058 (4)
O1	0.0641 (17)	0.111 (2)	0.080 (2)	−0.0158 (16)	−0.0138 (15)	0.0255 (17)
O2	0.0459 (14)	0.121 (2)	0.097 (2)	−0.0142 (15)	0.0225 (14)	−0.0075 (18)
O3	0.0360 (11)	0.0520 (12)	0.0340 (10)	0.0031 (9)	0.0067 (8)	−0.0017 (9)
O4	0.0971 (19)	0.0564 (15)	0.0523 (14)	−0.0270 (14)	0.0186 (13)	−0.0149 (12)
O5	0.0910 (18)	0.0397 (13)	0.0527 (14)	−0.0054 (12)	0.0057 (12)	0.0067 (11)
N1	0.0494 (17)	0.0518 (18)	0.072 (2)	−0.0018 (14)	0.0060 (16)	0.0078 (15)
C1	0.0341 (17)	0.052 (2)	0.062 (2)	−0.0015 (14)	0.0099 (14)	0.0064 (16)
C2	0.0455 (18)	0.0397 (17)	0.0544 (19)	0.0011 (14)	0.0196 (15)	0.0049 (15)
C3	0.0478 (18)	0.0326 (16)	0.0416 (16)	−0.0009 (13)	0.0106 (13)	0.0023 (13)
C4	0.066 (2)	0.0406 (18)	0.0448 (18)	−0.0007 (16)	0.0217 (16)	0.0034 (14)
C5	0.074 (2)	0.0439 (18)	0.0320 (16)	0.0014 (17)	0.0050 (16)	−0.0006 (14)
C6	0.060 (2)	0.050 (2)	0.0435 (18)	0.0034 (16)	−0.0073 (15)	−0.0039 (15)
C7	0.0419 (17)	0.0498 (19)	0.0401 (16)	0.0021 (14)	0.0038 (13)	−0.0024 (14)
C8	0.0437 (17)	0.0301 (15)	0.0338 (15)	0.0027 (13)	0.0071 (12)	0.0016 (12)
C9	0.0373 (16)	0.0442 (17)	0.0408 (16)	0.0020 (13)	0.0078 (12)	0.0006 (13)
C10	0.0316 (15)	0.0443 (18)	0.0398 (16)	−0.0006 (13)	0.0068 (12)	−0.0044 (13)
C11	0.0441 (18)	0.0423 (18)	0.0490 (19)	−0.0011 (14)	0.0135 (14)	−0.0017 (15)
C12	0.097 (3)	0.044 (2)	0.086 (3)	−0.006 (2)	0.010 (2)	0.014 (2)
C13	0.0477 (18)	0.0420 (18)	0.0390 (16)	−0.0032 (14)	0.0070 (13)	−0.0064 (14)
C14	0.0535 (19)	0.0444 (18)	0.0364 (16)	−0.0044 (15)	−0.0019 (14)	−0.0008 (14)
C15	0.066 (2)	0.047 (2)	0.0475 (18)	0.0009 (17)	0.0032 (16)	−0.0035 (16)
C16	0.091 (3)	0.046 (2)	0.066 (2)	0.004 (2)	−0.005 (2)	−0.0083 (18)
C17	0.113 (4)	0.039 (2)	0.079 (3)	−0.018 (2)	−0.009 (3)	0.007 (2)
C18	0.102 (3)	0.061 (3)	0.061 (2)	−0.030 (2)	0.011 (2)	0.007 (2)
C19	0.075 (2)	0.054 (2)	0.0432 (18)	−0.0142 (18)	0.0092 (16)	−0.0003 (16)

### (II) Methyl (E)-2-({4-chloro-2-[(E)-2-nitrovinyl]phenoxy}methyl)-3-(2-chlorophenyl)acrylate Geometric parameters (Å, º)

**Table:**

Cl1—C19	1.734 (4)	C7—H7	0.9300
Cl2—C5	1.749 (3)	C9—C10	1.488 (4)
O1—N1	1.220 (4)	C9—H9A	0.9700
O2—N1	1.215 (4)	C9—H9B	0.9700
O3—C8	1.365 (3)	C10—C13	1.336 (4)
O3—C9	1.448 (3)	C10—C11	1.491 (4)
O4—C11	1.198 (4)	C12—H12A	0.9600
O5—C11	1.328 (4)	C12—H12B	0.9600
O5—C12	1.440 (4)	C12—H12C	0.9600
N1—C1	1.444 (4)	C13—C14	1.467 (4)
C1—C2	1.332 (4)	C13—H13	0.9300
C1—H1	0.9300	C14—C19	1.393 (4)
C2—C3	1.442 (4)	C14—C15	1.396 (4)
C2—H2	0.9300	C15—C16	1.374 (5)
C3—C4	1.409 (4)	C15—H15	0.9300
C3—C8	1.410 (4)	C16—C17	1.372 (6)
C4—C5	1.365 (5)	C16—H16	0.9300
C4—H4	0.9300	C17—C18	1.364 (6)
C5—C6	1.367 (5)	C17—H17	0.9300
C6—C7	1.379 (4)	C18—C19	1.374 (5)
C6—H6	0.9300	C18—H18	0.9300
C7—C8	1.378 (4)		
			
C8—O3—C9	116.6 (2)	H9A—C9—H9B	108.4
C11—O5—C12	117.3 (3)	C13—C10—C9	124.4 (3)
O2—N1—O1	122.4 (3)	C13—C10—C11	117.5 (3)
O2—N1—C1	119.8 (3)	C9—C10—C11	118.0 (2)
O1—N1—C1	117.8 (3)	O4—C11—O5	123.3 (3)
C2—C1—N1	119.1 (3)	O4—C11—C10	124.9 (3)
C2—C1—H1	120.4	O5—C11—C10	111.8 (3)
N1—C1—H1	120.4	O5—C12—H12A	109.5
C1—C2—C3	129.5 (3)	O5—C12—H12B	109.5
C1—C2—H2	115.3	H12A—C12—H12B	109.5
C3—C2—H2	115.3	O5—C12—H12C	109.5
C4—C3—C8	117.4 (3)	H12A—C12—H12C	109.5
C4—C3—C2	117.5 (3)	H12B—C12—H12C	109.5
C8—C3—C2	125.1 (3)	C10—C13—C14	126.8 (3)
C5—C4—C3	120.8 (3)	C10—C13—H13	116.6
C5—C4—H4	119.6	C14—C13—H13	116.6
C3—C4—H4	119.6	C19—C14—C15	117.3 (3)
C4—C5—C6	121.1 (3)	C19—C14—C13	120.9 (3)
C4—C5—Cl2	119.7 (3)	C15—C14—C13	121.8 (3)
C6—C5—Cl2	119.2 (3)	C16—C15—C14	121.1 (3)
C5—C6—C7	119.5 (3)	C16—C15—H15	119.5
C5—C6—H6	120.2	C14—C15—H15	119.5
C7—C6—H6	120.2	C17—C16—C15	120.2 (4)
C8—C7—C6	120.9 (3)	C17—C16—H16	119.9
C8—C7—H7	119.6	C15—C16—H16	119.9
C6—C7—H7	119.6	C18—C17—C16	120.0 (3)
O3—C8—C7	123.9 (2)	C18—C17—H17	120.0
O3—C8—C3	115.9 (2)	C16—C17—H17	120.0
C7—C8—C3	120.2 (3)	C17—C18—C19	120.3 (4)
O3—C9—C10	108.2 (2)	C17—C18—H18	119.8
O3—C9—H9A	110.1	C19—C18—H18	119.8
C10—C9—H9A	110.1	C18—C19—C14	121.1 (3)
O3—C9—H9B	110.1	C18—C19—Cl1	119.3 (3)
C10—C9—H9B	110.1	C14—C19—Cl1	119.5 (3)
			
O2—N1—C1—C2	11.6 (5)	O3—C9—C10—C11	81.2 (3)
O1—N1—C1—C2	−169.3 (3)	C12—O5—C11—O4	−1.3 (5)
N1—C1—C2—C3	−179.8 (3)	C12—O5—C11—C10	178.1 (3)
C1—C2—C3—C4	−175.6 (3)	C13—C10—C11—O4	−2.9 (5)
C1—C2—C3—C8	5.8 (5)	C9—C10—C11—O4	173.0 (3)
C8—C3—C4—C5	−0.5 (4)	C13—C10—C11—O5	177.7 (3)
C2—C3—C4—C5	−179.3 (3)	C9—C10—C11—O5	−6.3 (4)
C3—C4—C5—C6	−0.4 (5)	C9—C10—C13—C14	5.3 (5)
C3—C4—C5—Cl2	179.1 (2)	C11—C10—C13—C14	−179.0 (3)
C4—C5—C6—C7	1.0 (5)	C10—C13—C14—C19	−137.2 (3)
Cl2—C5—C6—C7	−178.5 (2)	C10—C13—C14—C15	45.7 (4)
C5—C6—C7—C8	−0.5 (5)	C19—C14—C15—C16	1.1 (5)
C9—O3—C8—C7	−10.8 (4)	C13—C14—C15—C16	178.3 (3)
C9—O3—C8—C3	168.6 (2)	C14—C15—C16—C17	0.0 (5)
C6—C7—C8—O3	178.8 (3)	C15—C16—C17—C18	−0.6 (6)
C6—C7—C8—C3	−0.5 (4)	C16—C17—C18—C19	0.0 (6)
C4—C3—C8—O3	−178.3 (2)	C17—C18—C19—C14	1.1 (6)
C2—C3—C8—O3	0.3 (4)	C17—C18—C19—Cl1	−179.1 (3)
C4—C3—C8—C7	1.0 (4)	C15—C14—C19—C18	−1.7 (5)
C2—C3—C8—C7	179.7 (3)	C13—C14—C19—C18	−178.9 (3)
C8—O3—C9—C10	−167.7 (2)	C15—C14—C19—Cl1	178.6 (2)
O3—C9—C10—C13	−103.1 (3)	C13—C14—C19—Cl1	1.4 (4)

### (II) Methyl (E)-2-({4-chloro-2-[(E)-2-nitrovinyl]phenoxy}methyl)-3-(2-chlorophenyl)acrylate Hydrogen-bond geometry (Å, º)

**Table:**

*D*—H···*A*	*D*—H	H···*A*	*D*···*A*	*D*—H···*A*
C6—H6···O4^i^	0.93	2.56	3.371 (4)	146
C7—H7···O2^ii^	0.93	2.58	3.476 (4)	161
C18—H18···O1^iii^	0.93	2.60	3.485 (5)	160

Symmetry codes: (i) *x*−1/2, −*y*+1/2, *z*−1/2; (ii) *x*−1, *y*, *z*; (iii) −*x*+1, −*y*, −*z*+2.
